# Anion Exchange Chromatography–Mass Spectrometry to Characterize Proteoforms of Alpha-1-Acid Glycoprotein during and after Pregnancy

**DOI:** 10.1021/acs.jproteome.4c00107

**Published:** 2024-06-19

**Authors:** Guusje van Schaick, Manfred Wuhrer, Constantin Blöchl, Radboud J. E. M. Dolhain, Elena Domínguez-Vega

**Affiliations:** † Center for Proteomics and Metabolomics, Leiden University Medical Center, Albinusdreef 2, 2333 ZA Leiden, The Netherlands; ‡ Department of Rheumatology, Erasmus Medical Center, Wytemaweg 80, 3015 CN Rotterdam, The Netherlands

**Keywords:** alpha-1-acid glycoprotein, anion exchange chromatography, mass spectrometry, glycosylation, pregnancy, proteoforms

## Abstract

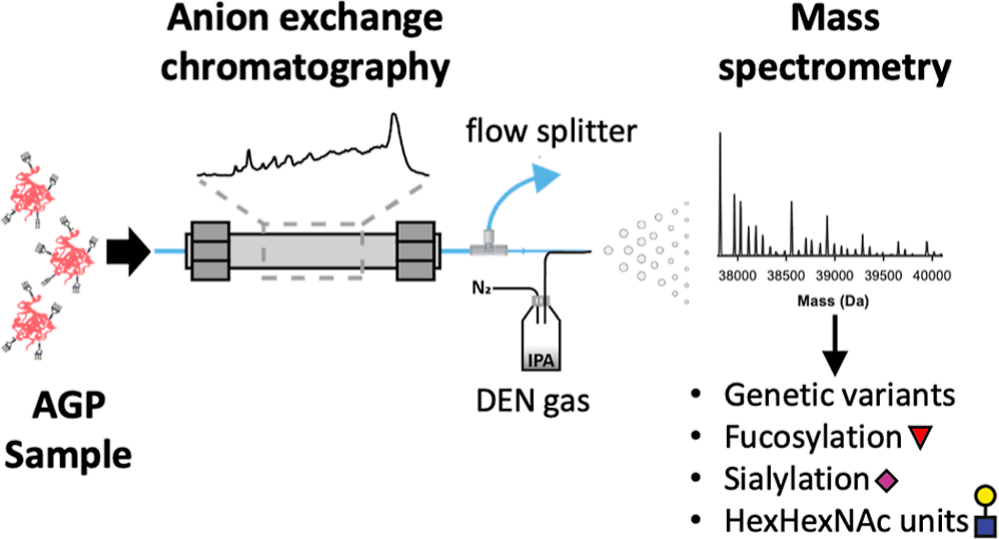

Alpha-1-acid glycoprotein (AGP) is a heterogeneous glycoprotein fulfilling key roles in many biological processes, including transport of drugs and hormones and modulation of inflammatory and immune responses. The glycoform profile of AGP is known to change depending on (patho)physiological states such as inflammatory diseases or pregnancy. Besides complexity originating from five N-glycosylation sites, the heterogeneity of the AGP further expands to genetic variants. To allow in-depth characterization of this intriguing protein, we developed a method using anion exchange chromatography (AEX) coupled to mass spectrometry (MS) revealing the presence of over 400 proteoforms differing in their glycosylation or genetic variants. More precisely, we could determine that AGP mainly consists of highly sialylated higher antennary structures with on average 16 sialic acids and 0 or 1 fucose per protein. Interestingly, a slightly higher level of fucosylation was observed for AGP1 variants compared to that of AGP2. Proteoform assignment was supported by integrating data from complementary MS-based approaches, including AEX–MS of an exoglycosidase-treated sample and glycopeptide analysis after tryptic digestion. The developed analytical method was applied to characterize AGP from plasma of women during and after pregnancy, revealing differences in glycosylation profiles, specifically in the number of antennae, HexHexNAc units, and sialic acids.

## Introduction

Alpha-1-acid glycoprotein (AGP) is involved in many biological processes, including the transport of basic and neutral molecules and the modulation of cell proliferation/differentiation.^1−3^ This acute-phase protein also plays a major role in immune and inflammatory responses, during which it is strongly upregulated. For instance, AGP concentrations increased to 167 mg/dl for COVID-19 patients compared to 69 mg/dl for healthy individuals.^4^

AGP exists as two genetic variants (AGP1 and AGP2) encoded by two adjacent genes. Although the variants differ only in 20 amino acids in sequence, physiological differences have been reported between them. The concentration of AGP1 in serum is between 3-fold and 100-fold higher compared to AGP2.^5,6^ Moreover, the binding sites are slightly different as AGP2 has a smaller binding site leading to different affinities for certain molecules.^2^ For example, imatinib binds weaker and less specific to AGP2.^7^ Furthermore, AGP2 has a free cysteine that can interact with other molecules or form a disulfide bond with a free cysteine (i.e., cysteinylation), which is not the case for AGP1.^2,8^ Finally, AGP1 is polymorphic giving rise to three genetic variants (AGP1*F1, AGP1*F2, and AGP1*S) that only differ in sequence by a few amino acids.^5,8^ While the AGP1*F1 and AGP1*S variants are observed worldwide, the AGP1*F2 variant is found in European populations.^6^

Besides genetic variants, glycosylation is responsible for the high proteoform heterogeneity of AGP. The mass of five complex-type N-glycans present in AGP contributes to around 45% of the total molecular mass of AGP.^9^ Most glycans consist of highly sialylated di-, tri-, or tetra-antennary structures.^5,10^ Interestingly, changes in the glycoform profile have been associated with various physiological and pathophysiological conditions, including inflammation, pregnancy, severe rheumatoid arthritis, liver cirrhosis, hepatitis, asthma, and cancer.^10−12^ For instance, in early stage acute-phase reactions, the degree of branching decreases, while fucosylation increases.^13^ During pregnancy, the branching of AGP glycans increased, accompanied by a decrease in the degree of fucosylation.^14,15^ Moreover, the presence of multifucosylated tetra-antennary glycans has been suggested as a potential diagnostic marker for hepatocellular carcinoma.^16^ The glycosylation profile of AGP may also serve as a prognostic tool as increased branching and lower sialylation were observed in individuals who are at higher risk of developing type 2 diabetes.^17^ This motivates the development of analytical tools to investigate the proteoform profile of the AGP that may then be connected to specific functions and diseases.

Currently, most analytical strategies to determine the glycan heterogeneity of AGP are based on released glycan or glycopeptide approaches.^13,17,18^ Although these techniques are excellent tools for obtaining an overview of the sheer complexity of AGP glycosylation, information on the coexisting glycans, as well as on the genetic variants, is lost. To address this challenge, an intact protein analysis approach has been proposed using direct infusion mass spectrometry (MS).^8^ While this method improved the proteoform assignment, new difficulties were encountered as glycoforms close in mass overlapped in the mass spectrum, hampering confident assignment of glycan compositions regarding fucosylation and sialylation levels. A charge-based separation approach prior to MS detection, such as capillary zone electrophoresis (CZE)^19,20^ or ion exchange chromatography, could separate proteoforms based on sialic acids and thereby enable more confident glycoform identification.

**Figure 1:**
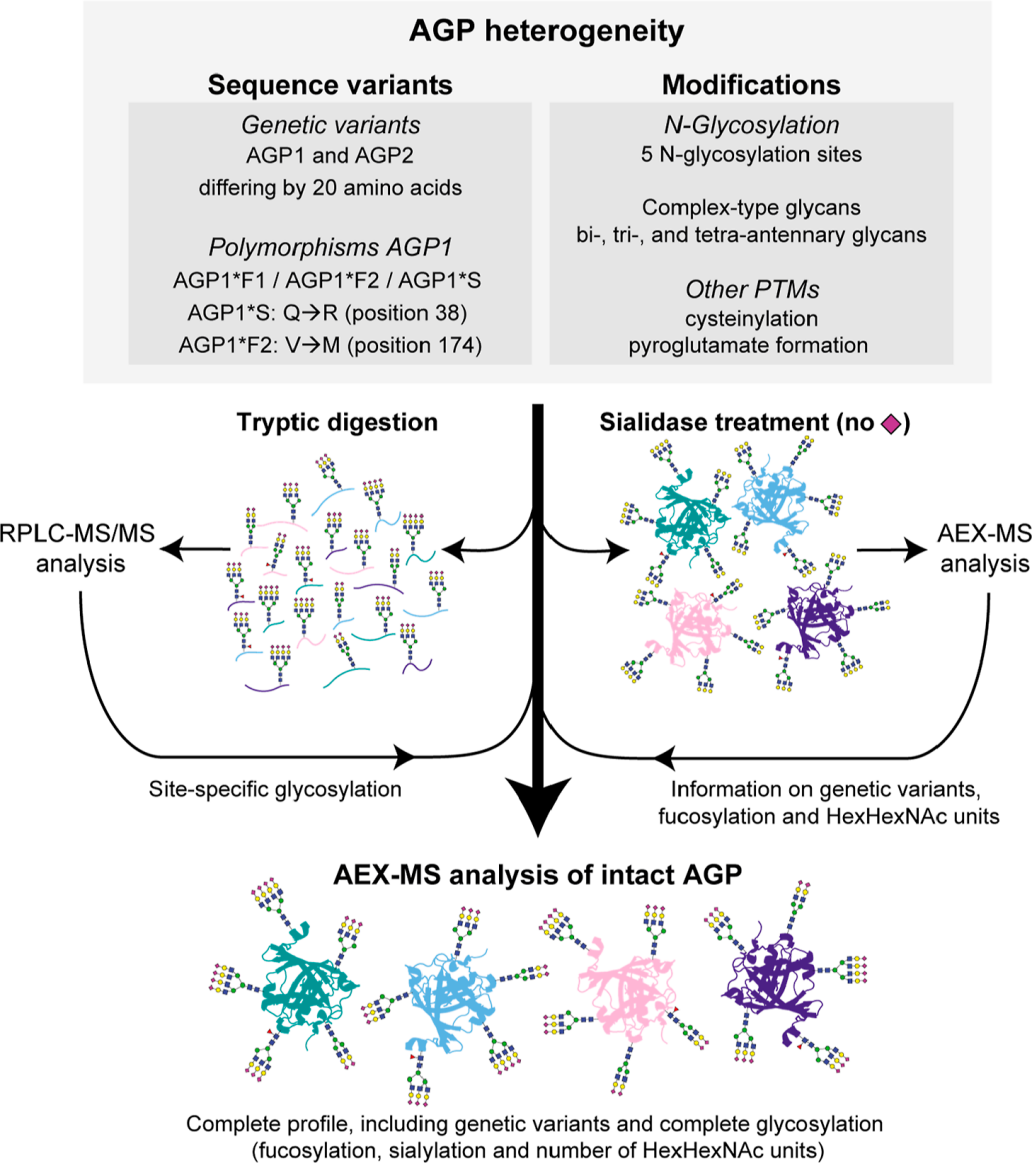
Overview of the heterogeneity of AGP and the strategy of the characterization. The complete AGP heterogeneity, including the different genetic variants and extensive N-glycosylation, was investigated on an intact level with AEX–MS. Due to the sheer complexity of the intact mass spectra, an integrated approach was used, where results of glycopeptide analysis and analysis after sialidase treatment were used for confident assignment. For each of the analysis methods, the obtained information is indicated.

In this study, we aimed to address challenges that arise from intact AGP heterogeneity during analysis to improve the in-depth characterization, resulting in not only the assignment of more proteoforms but also allowing relative quantification of detected species. To improve AGP proteoform resolution and identification, we implemented an MS-conjugated anion exchange chromatography (AEX) method—previously applied to characterize the glycoproteins ovalbumin,^21^ erythropoietin,^22^ and Myozyme.^23^ This method allowed us to distinguish variants despite their similar sequences and to assign glycan combinations that are close in mass on an intact level (for example, glycoforms with an additional sialic acid or two fucose units), highlighting the need for upfront separations before MS detection. Moreover, minimal sample treatment is required prior to analysis. Proteoform assignment was supported by bottom-up glycopeptide analysis and AEX–MS analysis of AGP treated with a sialidase (Figure 1). Subsequently, we applied the AGP AEX–MS method to study the changes in proteoforms during and after pregnancy.

## Experimental Section

### Materials and Samples

Ammonium formate (>97.0%) was obtained from Fluka (Steinheim, Germany). Formic acid (FA) (>98.0%) and 2-propanol (IPA) were purchased from Riedel-De Haen (Seelze, Germany). Ammonium bicarbonate (ABC), dithiothreitol (DTT), iodoacetamide (IAA), and tris(hydroxymethyl)aminomethane (≥99.8%) were obtained from Sigma-Aldrich (Zwijndrecht, The Netherlands). Trifluoroacetic acid (TFA) was purchased from Merck (Darmstadt, Germany). Acetonitrile (ACN) was purchased from Biosolve (Valkenswaard, The Netherlands). Milli-Q water was provided by Purelab ultra (ELGA Labwater, Ede, The Netherlands). The AGP standard material (≥95%; purified from human plasma) used for method development was purchased from Athens Research & Technology (Georgia, USA).

The treatment of the glycans of the AGP standard was performed with SialEXO obtained from Genovis (Lund, Sweden). The AGP standard (1 μg/μL) was buffer exchanged to a 20 mM Tris buffer (pH 6.8) using 10 kDa Vivaspin MWCO filters from Sartorius (Göttingen, Germany). Thereafter, SialEXO was added, as indicated in the producer specifications. The sample was incubated overnight at 37 °C. Finally, the samples were buffer exchanged with 50 mM ammonium formate (pH 5.5) and analyzed with AEX–MS.

### Capturing of AGP from Plasma

The (pregnant) women samples were previously studied, and detailed information on the samples can be found elsewhere.^24^ From the available plasma samples, two samples from four healthy women were selected in their second trimester and three months postpartum. The AGP protein in these samples was captured from 100 μL of plasma using AGP capture select beads (Life Technologies/Thermo scientific). The samples were diluted four times before they were applied to the beads. The mixture of the sample and beads was incubated for 1 h while shaking at moderate speed. The samples were first washed three times with 1× PBS (prepared by dilution of 5× PBS solution composed of 28.5 mg/mL Na_2_HPO_4_·2H_2_O, 2.4 mg/mL KH_2_PO_4_, and 42.5 mg/mL NaCl dissolved in Milli-Q water). Subsequently, the samples were washed three times with Milli-Q water to remove the salts. The elution was performed by the addition of 500 mM TFA, incubation for 10 min while shaking at moderate speed, and centrifugation for 1 min at 500 rpm. Finally, the samples were dried by vacuum centrifugation and reconstituted in 50 mM ammonium formate (pH 5.5), followed by analysis with AEX–MS.

### Glycopeptide Analysis of AGP Using RPLC-MS/MS

Prior to tryptic digestion, the pooled AGP sample was diluted with a reducing SDS-PAGE gel. The sample (5 μL; 1 μg/μL) was mixed with 13 μL of Milli-Q water, and 6 μL of Laemmli buffer (4×) containing mercaptoethanol was added to the solution. The sample was heated for 10 min at 100 °C. After loading 20 μL of the sample on a NuPage 4–12% Bis-Tris 10-well gel (Invitrogen), the gel was run using an MES buffer (20× diluted; Novex) for 40 min at 200 V. Finally, the gel was washed three times with Milli-Q water followed by staining with SimplyBlue SafeStain (Invitrogen). The sample was run in duplicate on the gel, and both bands were used as separate samples for the glycopeptide analysis.

Next, the AGP band was cut from the gel and further processed. The gel pieces were added in a tube together with 500 μL of ACN and incubated for 10 min. Subsequently, the gel pieces were incubated with 500 μL of 50% ACN in 100 mM NH_4_HCO_3_ and 500 μL of ACN (both 10 min). After each step, the remaining liquid was removed prior to addition of the next solution. The samples were reduced with 10 mM DTT for 20 min at 60 °C followed by alkylation with 55 mM IAA for 20 min at room temperature. After washing the samples with 25 mM ABC buffer and ACN, trypsin was added in two steps: first 30 μL (12.8 ng/μL) of enzyme followed by another 20 μL of enzyme. The samples were digested overnight at 37 °C. Finally, the peptides were extracted using a mixture of Milli-Q water, ACN, and FA (ratio 50:50:1) and freeze-dried followed by storage at −20 °C.

AGP tryptic peptides were dissolved in 95/3/0.1 v/v/v Milli-Q water/ACN/FA and analyzed by online C18 nano-HPLC MS/MS with a system consisting of an Easy nLC 1000 gradient HPLC system (Thermo, Bremen, Germany) and a LUMOS mass spectrometer (Thermo). Samples were injected onto a homemade precolumn (100 μm × 15 mm; Reprosil-Pur C18-AQ 3 μm, Dr. Maisch, Ammerbuch, Germany) and eluted via a homemade analytical nano-HPLC column (30 cm × 50 μm; Reprosil-Pur C18-AQ 3 μm). The gradient was run from 2 to 40% solvent B (20/80/0.1 Milli-Q water/ACN/FA v/v/v) in 30 min. The nano-HPLC column was drawn to a tip of ∼10 μm and acted as the electrospray needle of the MS source. The LUMOS mass spectrometer was operated in data-dependent MS/MS mode for a cycle time of 3 s, with an HCD collision energy at 35% and recording of the MS2 spectrum in the Orbitrap. In the master scan (MS1), the resolution was 120,000, and the scan range was 400–2500 at an automatic gain control (AGC) target of the “standard”. A lock mass correction on the background ion *m*/*z* = 445.12003 was used. Dynamic exclusion after *n* = 1 with an exclusion duration of 10 s was used. Charge states 1–7 were included. For MS2 precursors, they were isolated with the quadrupole with an isolation width of 1.2 Da. The MS scan range was 190–2000. The MS2 scan resolution was 30,000 with an AGC target of the “standard” with a maximum fill time of “auto”. An additional MS2 scan was triggered if HexNAc oxonium ion 204.087 was present in an MS2 spectrum. The same precursor was again selected, now with a stepped collision energy of 25, 32, and 39%, and the maximum fill time was adjusted to 100 ms at a normalized AGC target of 200%, a mass resolution of 30,000, and a mass range of 150–3500.

### AEX Coupled to MS

The AEX measurements were performed using a biocompatible Ultimate 3000 instrument (Thermo Fisher Scientific) with a ProPac SAX-10 column (2.0 mm × 250 mm, 10 μm; Thermo Fisher Scientific). After evaluation of several mobile phases, the optimal mobile phase for the intact AGP was 50 mM ammonium formate at pH 5.5 (A) and 200 mM FA at pH 2.5 (B). Mobile phase A was prepared by diluting a 200 mM stock solution of ammonium formate to 50 mM followed by evaluation of the pH using a pH meter and adjustment of the pH to 5.5 using 50 mM FA. Mobile phase B consisted of 200 mM FA without pH adjustment. The gradient linearly increased from 0 to 100% B in 45 min. The mobile phases used for exoglycosidase-treated AGP were 10 mM ammonium acetate + 10 mM ammonium formate at pH 6.5 (A) and 10 mM acetic acid + 10 mM FA at pH 3.0 (B). These mobile phases were prepared by mixing solutions of 10 mM ammonium acetate and 10 mM ammonium formate (A) and 10 mM acetic acid and 10 mM FA (B). The pH did not need further adjustment after the mixing. The separation was accomplished by a linear gradient from 0 to 100% B in 45 min. Both methods included a column cleaning step at 100% B (5 min) and re-equilibrated at 0% B (20 min) at the end of the method. The flow rate, column temperature, and UV wavelength were 0.25 mL/min, 25 °C, and 280 nm, respectively. The injected amount was 15 μL for AEX-UV analyses and 30 μL for AEX–MS analyses.

The AEX separation was online coupled to a Bruker 15T solariX Fourier-transform ion cyclotron resonance (FTICR)-MS system (Bruker Daltonics, Bremen, Germany) operated in positive-ion mode. The hyphenation of AEX and MS was performed by a post-separation splitter reducing the flow around 100 times and by enrichment of the nitrogen gas with IPA. The ESI capillary voltage was 1100 V, and the end plate offset was −500 V. The nebulizer gas flow rate was 0.4 bar, the dry gas flow rate was 3 L/min, and the dry gas temperature of the nitrogen was 220 °C. The ion funnel 1 was set at 180 V, radiofrequency amplitude at 300 V_pp_, and skimmer 1 at 135 V. The in-source collision energy was 40 V, and the collision voltage in the collision cell was set to −15 V. The acquisition range was *m*/*z* 796.9–8000. The resolution obtained using these conditions was 33,000 at 400 *m*/*z*. The accumulation time was set to 1 s, and the data acquisition size was set to 64,000 data points. The final mass spectrum was obtained by the summation of 10 spectra.

### Data Analysis

The assignment of AGP proteoforms in the AEX–MS measurements was done manually using Data Analysis from Bruker Daltonics. The maximum entropy algorithm was used for charge state deconvolution. After evaluation, the resolution was set at 4000, and data spacing was 1.0 point, providing good quality of the deconvoluted mass spectra. The base peak chromatograms (BPCs) and extracted ion chromatograms (EICs) were smoothed with the Gauss smoothing algorithm (1 cycle) for visualization purposes. For the calculation of the glycan compositions, average masses were used, including hexose (H, 162.14 Da), fucose (F; 146.14 Da), *N*-acetylhexosamine (N, 203.20 Da), and *N*-acetylneuraminic acid/sialic acid (S; 291.26 Da). The area of EICs of AGP proteoforms was obtained using the software Skyline^25^ (version 23.1). The EICs were generated with the most abundant charge states (i.e., 8+ and 9+). For these charge states, the most abundant isotope (automatically calculated by software) was used. For the confident assignment, the expected retention time and the differences between theoretical and experimental masses were considered. The difference between the theoretical and experimental masses is presented in Tables S1–S3 for the different samples. After manual integration of the AGP glycoform peak in the EICs, the obtained areas were used to perform relative quantification, i.e., normalizing the areas to 100% for each genetic variant.

## Results and Discussion

### Development of an AEX–MS Method for AGP Characterization

Over the years, MS-compatible AEX methods shifted from elution with increasing salt concentration to elution by altering the pH (pH gradient), allowing the use of lower salt concentrations and, thereby, improved MS compatibility.^26^ A key parameter for pH gradient AEX separation is the isoelectric point(s) (pI) of the proteins. The elution from the stationary phase is promoted when the pH of the mobile phase approaches the pI.^27^ The theoretical pI of the AGP backbone is between 5.0 and 5.1 depending on the genetic variant (for the sequences of the variants, see Figure S1). Nevertheless, the AGP carries heavily sialylated glycans on its five N-glycosylation sites,^9^ leading to drastically decreased pI values after including the glycosylation (pI values between 2.8 and 3.8).^5^

Previous AEX–MS applications showed good separation of intact proteins with relatively low pI values, including erythropoietin and prolyl-alanyl-specific endoprotease, using a pH gradient from 5.5 to 2.5 with between 30 and 50 mM ammonium formate as buffer.^22,28^ When applying this method to a standard AGP sample, only the least acidic proteoforms eluted, while the rest of the proteoforms remained attached to the column (Figure S2a). Increasing the salt concentration of the mobile phases may lead to lower retention and, therefore, could benefit AGP analysis. While some proteoforms started to elute earlier after increasing ionic strength to 100 mM ammonium formate, the majority still eluted during column washing (Figure S2b). A salt concentration of 150 mM resulted in elution of all proteoforms before 20 min but without improving the separation quality (Figure S2c), suggesting that solely a pH gradient was not sufficient to achieve elution. Therefore, we explored the potential of a combined salt and pH gradient, where the decrease in pH is accompanied by an increase in ionic strength.^29^ By performing a gradient using mobile phases composed of 50 mM ammonium formate at pH 5.5 (A) and 200 mM FA at pH 2.5 (B) (Figure S2d), good retention and elution of AGP proteoforms were obtained. Around ten (partly) resolved peaks could be distinguished in the chromatogram in an elution window from 10 to 60 min.

Since an MS-compatible buffer composed of ammonium formate was used, the AEX separation was online coupled to the mass spectrometer, enabling direct proteoform characterization of the eluting peaks. To ensure high-quality MS data, a hyphenation strategy was used consisting of a decrease in flow (via flow splitter) enabling nano-electrospray ionization (ESI) and the use of dopant-enriched nitrogen (DEN) gas in the source. The postcolumn splitter reduced the flow 100 times (i.e., from 250 to 2.5 μL/min), which allowed online coupling to the nano-ESI source. Our group recently showed that the use of DEN gas (specifically IPA-enriched nitrogen gas) greatly improved the spectral quality of highly glycosylated proteins that normally suffer from low ionization efficiency.^30^ A similar phenomenon was observed for AGP, where IPA-DEN gas provided higher signal intensity and good quality spectra enabling proteoform assignment (Figure S3). In the obtained BPC, the first peaks (up to 30 min separation time) are more distinct, whereas the following peaks (between 30 and 48 min) are broader (Figure S2e). The phenomenon of peak broadening along the salt-mediated pH gradient can be attributed to the change from low ionic strength mobile phases (generally focusing chromatographic peaks) to higher ionic strength (broadening the peaks).^27^ Using the obtained mass spectra of each chromatographically separated peak, we could detect a plethora of different masses below, which corresponded to AGP proteoforms.

### Proteoform Characterization of Pooled AGP

While protein masses can be retrieved from highly complex spectra, assignment benefits from the data integration of orthogonal or complementary analytical approaches (Figure 1). We used glycopeptide data to determine the site-specific glycosylation and glycan-trimming approaches to evaluate the level of different genetic variants. For the in-depth characterization, we used a commercially available standard AGP sample that was enriched from pooled human plasma. Since this sample is pooled from different healthy donors, it should contain different genetic variants and thereby provide a good overview of prevalent AGP proteoforms.

Using glycopeptide analysis, the glycan distribution and occupancy were investigated per glycosylation site. AGP has five known N-glycosylation sites at positions Asn15, Asn38, Asn54, Asn75, and Asn85 (Figure S1) that carry complex-type N-glycans.^5,10^ In the glycopeptide data, glycans were detected ranging from diantennary to highly branched glycans (up to glycoforms with H9N8) (Figure S4). All sites were fully occupied with glycans, except for site Asn54 where a very minor amount of nonglycosylated peptide was found (0.08%). Almost all detected glycans were sialylated, where the majority of glycans carried two to three sialic acids, leading to an estimated average sialylation level of between 12 and 13 sialic acids per protein. Finally, glycans with no or one fucose were observed with higher levels for afucosylated species. These findings were in agreement with previous glycopeptide analyses of AGP.^13,18^ Besides the glycosylation heterogeneity, the presence of genetic variants also vastly contributes to protein heterogeneity. Interestingly, differences in the glycosylation profile between AGP1 and AGP2 could be observed for some of the sites (e.g., a slightly higher level of fucosylation was detected for AGP2 compared to AGP1 for site Asn85). The glycopeptides of sites Asn38 and Asn54 were identical for AGP1 and AGP2, precluding the differentiation of these two variants with regard to the glycosylation on those sites. Moreover, the obtained glycopeptide of site Asn15 is the same for the AGP1*S and AGP2 variants. Altogether, the assessment of glycosylation differences of the different genetic variants is restricted when only glycopeptides are monitored, yet the information obtained is highly valuable for annotation of the spectra of the intact AGP.

**Figure 2:**
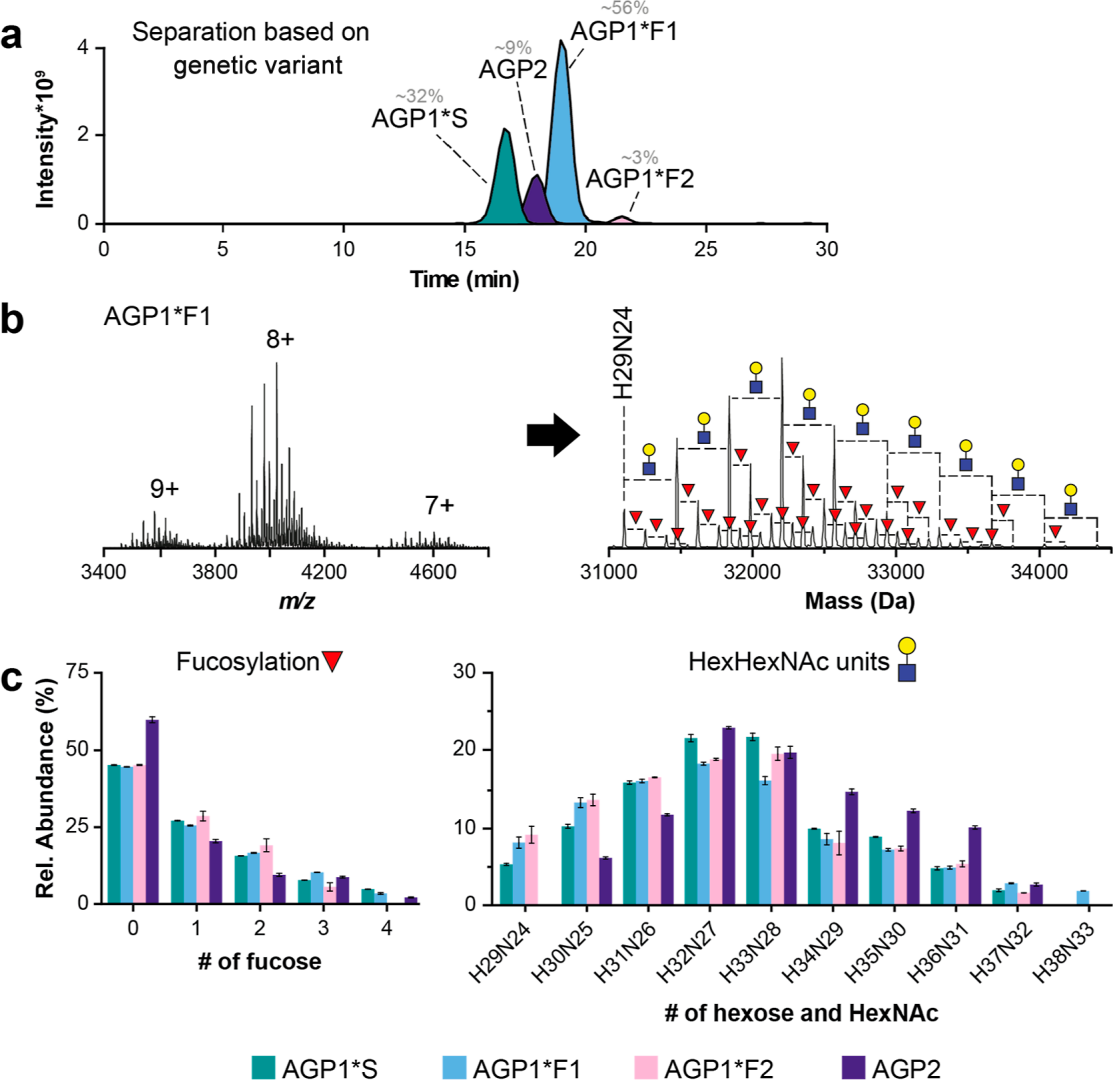
Information on the genetic variants and their glycosylation (HexHexNAc units and fucosylation) was obtained with the AEX–MS analysis of AGP after sialidase treatment. The mobile phases were composed of 10 mM ammonium acetate with 10 mM ammonium formate at pH 6.5 (A) and 10 mM acetic acid with 10 mM FA at pH 3.0 (B). (a) EICs of the three most abundant glycoforms of each genetic variant, where AGP1*S is presented in green, AGP1*F1 in blue, AGP1*F2 in pink, and AGP2 in purple. (b) Mass spectrum of the AGP1*F1 chromatographic peak, including the charge states (left) and the deconvoluted mass spectrum (right). (c) Relative quantification of the assigned proteoforms of the AGP standard subjected to sialidase treatment. Both the level of fucosylation and the number of HexHexNAc units were determined for the different genetic variants. The samples were measured in duplicate, and the error bars indicate the deviation between the replicates. The complete list of assigned glycoforms, including their abundances, can be found in Table S1.

To assign the masses detected with AEX–MS to AGP proteoforms next to glycopeptides, the analysis of the AGP standard sample was performed after sialidase treatment. The AEX separation of the desialylated sample provided a single peak comprising many different proteoforms. This complete loss of proteoform separation (Figure S5) revealed that the number of sialic acids was the main contributor to the AEX separation of AGP. We adapted the AEX method to allow evaluation of the abundance of genetic variants in this sample without the contribution of sialic acids. Good separation of genetic variants was obtained using mobile phases composed of 10 mM ammonium acetate with 10 mM ammonium formate at pH 6.8 (A) and 10 mM FA with 10 mM acetic acid at pH 2.9 (B) (Figure 2). After analysis of the pooled AGP material, all four genetic variants were (partly) separated, most probably caused by a slightly higher pI for AGP1*S and AGP2 due to the presence of an additional arginine in the sequence. AGP1 (the sum of the three forms) was found to be around 10-fold more abundant than AGP2, while ratios between 3:1 and 5:1 are reported in literature.^6,31^ This ratio, however, can alter upon a changed physiological state.^32^ The most abundant form was the AGP1*F1 variant (around 56% of the total intensity corresponded to AGP1*F1 proteoforms), followed by AGP1*S (32% of the total intensity), AGP2 (9% of the total intensity), and finally AGP1*F2 (3% of the total intensity) (Figure 2a and Table S1). Additionally, the mass spectra of the desialylated AGP provided insights into some glycosylation features, such as the number of fucoses and HexHexNAc units specific for each of the genetic variants (Figure 2b). For all variants, the nonfucosylated glycoforms were most abundant (≥45% of all detected proteoforms), and up to four fucoses were detected per protein (Figure 2c; left panel). These findings are in agreement with previously performed RPLC-MS analysis of desialylated AGP.^8^ Moreover, we observed that the AGP2 variant showed slightly higher levels of nonfucosylated glycoforms compared to AGP1 variants, which could also be seen in a previous study^8^ (Figure 2c). A broad distribution of Hex and HexNAc units was observed with the composition of Hex_*n*_HexNAc_*n*–5_. The most abundant combination was H32N27 for AGP1*F1 and AGP1*S (20 and 22% of the assigned proteoforms), whereas H33N28 was the most abundant form for AGP1*F2 and AGP2 (20 and 23% of all assigned proteoforms) (Figure 2c; right panel). In addition to glycosylation, all proteoforms showed pyroglutamate formation at the N-terminal glutamine (decrease in mass of 17 Da) and the AGP2 proteoforms were cysteinylated at the cysteine of position 149 (mass increase of 119 Da). Cysteinylation of AGP2 was also previously reported by Bärenfänger et al. for the analysis of human AGP from pooled plasma.^8^ Overall, we were able to assign 142 proteoforms after desialylation, differing in genetic variant or glycosylation (Table S1).

**Figure 3:**
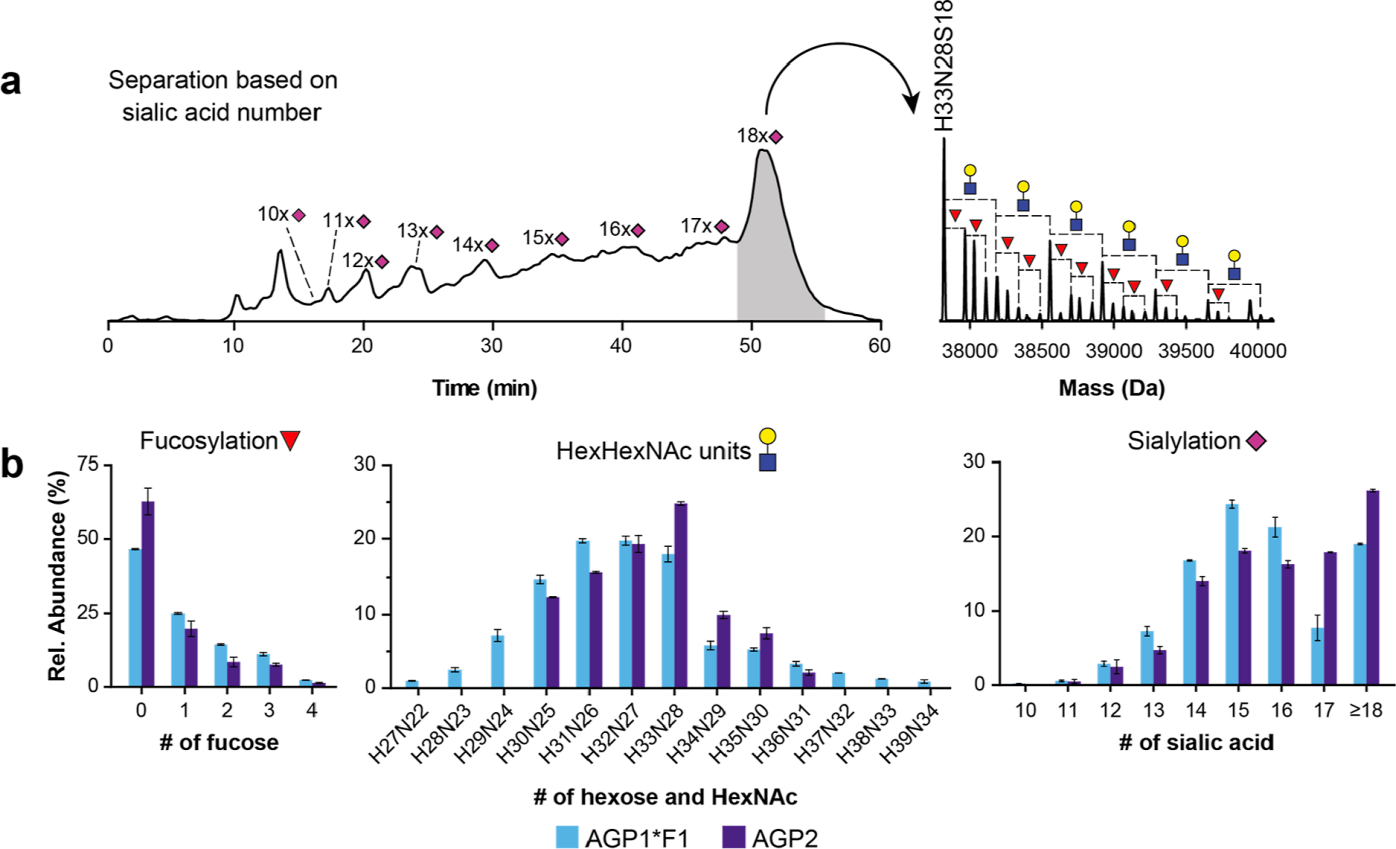
AEX–MS method for the separation, assignment, and quantification of AGP proteoforms. The mobile phases consisted of 50 mM ammonium formate at pH 5.5 (A) and 200 mM FA at pH 2.5 (B). (a) BPC of the complete AGP sample presented with the deconvoluted mass spectrum of the peak containing glycoforms with 18 sialic acids. (b) Relative quantification of assigned proteoforms of the AGP. The relative abundances were determined for the numbers of fucose units, HexHexNAc units, and sialic acids. A complete overview of the relative abundances per glycoform can be found in Table S2.

The next step was the assignment of the complete AGP proteoform profile. The AEX separation revealed information about the abundance of proteoforms with differences in the number of sialic acids. Within the elution window from 16 to 50 min, the sialic acid number increased from 10 to 18/19 sialic acids per protein (Figure 3a), with an average number of sialic acids between 15.5 and 16.0 (Figure 3b). A previous CZE-MS study also showed that all glycoforms were sialylated and that the lowest number of sialic acids was 10.^19^ This average sialic acid number is higher than that was predicted based on the glycopeptide data (that was an average of 12–13 sialic acids per protein). Underestimation of the abundance of highly sialylated glycopeptides due to an ionization bias of the glycopeptides is an often-occurring phenomenon that was previously observed for other highly sialylated glycoproteins, such as erythropoietin or SARS-CoV-2 receptor-binding domain protein.^33,34^ Furthermore, due to the ability of the AEX–MS method to separate proteoforms with different numbers of sialic acids, we could discriminate between species with two additional fucoses from the ones with one additional sialic acid (mass difference of 1 Da), increasing the confidence in our assignments (Figure S6a). Also, the H29N24F1S14 and H31N26S12 glycoforms could be distinguished based on retention time despite similarities in mass (differing by 2 Da in mass) (Figure S6b). Both intact RPLC-MS and direct infusion MS methods provide a fast overview of several AGP proteoforms but unfortunately cannot distinguish between these critical species.^8^ Using these criteria, we could determine the proteoforms that differ in fucose content or branching from the genetic variants within each sialic acid peak (Figure 3a). In total, we were able to assign 415 glycoforms in the pooled sample (Table S2). Due to low abundance of AGP1*S and AGP1*F2 proteoforms, we focused on the most abundant AGP1 form (AGP1*F1) and AGP2 for the relative quantification. Moreover, the level of sialylation, the number of HexHexNAc units, and the relative abundance of the two genetic variants were determined from the nonfucosylated glycoforms. The level of fucosylation was established based on the chromatographic peaks containing 11, 14, and 18 sialic acids as these peaks suffered the least from peak broadening (and thereby less peak overlap). The maximum species regarding Hex and HexNAc composition were H32N27 for AGP1*F1 and H33N28 for AGP2 (Figure 3b). The study of Ongay and Neusüß also described a degree of antennary for AGP2,^19^ but unfortunately no relative quantification was performed making direct comparison of the results difficult. Furthermore, the glycoforms without fucose were dominant, and this was more pronounced for AGP2 (63% afucosylation) compared to AGP1*F1 (47% afucosylation) (Figure 3b). The obtained results for the HexHexNAc distribution and degree of fucosylation were highly similar to the results for the samples without sialidase treatment.

### AEX–MS for Monitoring Pregnancy-Associated Changes in AGP Proteoforms

AGP glycosylation can be linked to many (patho)physiological states, including, but not limited, to different types of cancer and infections.^10−12^ Altered glycoform profiles, such as increased branching and decreased fucosylation, have been previously reported during pregnancies.^14,15^ To demonstrate the usefulness of our intact protein AEX–MS method for unraveling such associations, we analyzed the pregnancy-associated changes in AGP proteoforms from the plasma of healthy women.

We measured and compared plasma samples of four healthy women during the second trimester and samples taken after pregnancy (3 months postpartum). Approximately 100 μg of AGP was captured from 100 μL of plasma using AGP-specific affinity beads. The obtained AGP was dried and reconstituted in 35 μL of AEX mobile phase A, from which 30 μL was injected into the AEX column. To determine any potential proteoform-specific biases during the capture, the AGP standard sample was captured, analyzed, and compared with the profile without the capturing step. Similar separation profiles and comparable numbers of HexHexNAc units and fucoses were observed (Figure S7). Furthermore, the abundance of AGP1 and AGP2 variants was also similar (88% of the proteoforms of the non-captured sample were AGP1*F1, and this percentage was 89% for the captured sample), indicating that the capturing procedure had no apparent AGP proteoform bias.

**Figure 4:**
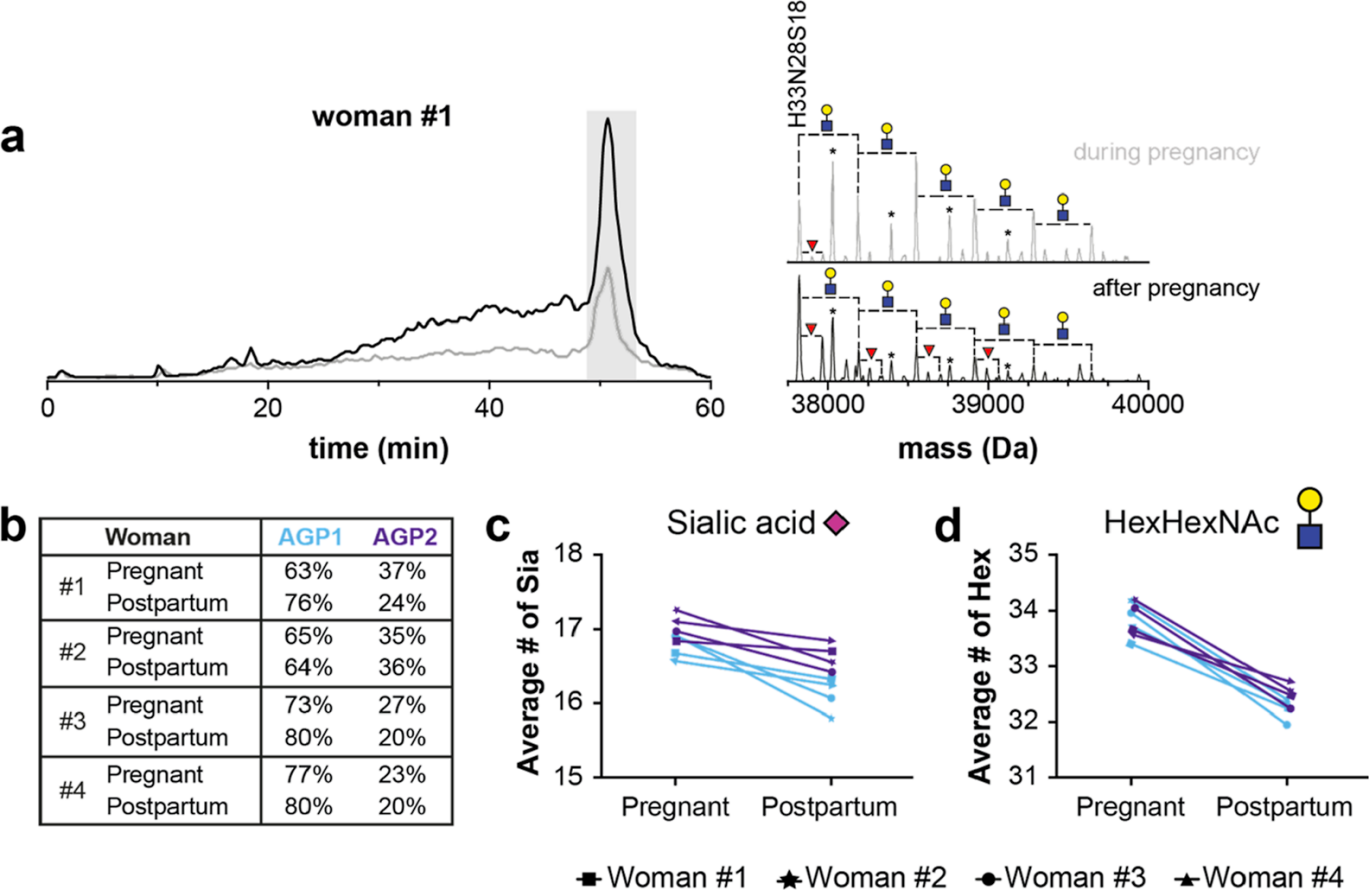
AEX–MS method for the separation, assignment, and quantification of AGP proteoforms of samples from (pregnant) women. (a) Example of BPCs obtained during the second trimester of pregnancy (gray trace) and after 3 months postpartum (black trace), including deconvoluted mass spectra of the peak containing glycoforms with 18 sialic acids. The AGP1*F1 glycoforms with differences in fucoses or HexHexNAc units are assigned in the mass spectrum, and the masses marked with asterisks are nonfucosylated glycoforms from AGP2. (b) Total relative abundance of AGP1*F1 and AGP2 glycoforms in the different samples. (c) Change in the average number of sialic acids during and after pregnancy. (d) Difference in the number of HexHexNAc units (presented as the average number of hexoses) during and after pregnancy. The blue lines represent the results obtained for AGP1*F1, and the purple lines represent the results of AGP2. For a complete overview of the relative abundances per glycoforms, see Table S3.

The profiles of AGP captured from plasma of the four women showed between 15.8 and 16.2 sialic acids per AGP molecule, similar to the AGP standard (15.5 to 16.0 sialic acids; Figures 4a and S8; Table S3). One of the investigated women (woman 3) had a heterozygous expression of AGP1 (both variants AGP1*F1 and AGP1*S, where the latter had a low abundance of around 6% of the total intensity of the detected proteoforms), whereas the other women (1, 2, and 4) appeared to be homozygous for AGP1*F1. In all samples, AGP2 glycoforms were detected in much lower amounts compared to AGP1 with abundances between 20 and 37% (Figure 4b). Similar to the pooled AGP standard sample, the average number of sialic acids per protein was slightly higher for the AGP2 glycoforms compared with the AGP1 forms (Figure 4c), i.e., on average, 16.1 sialic acids for AGP1 glycoforms and 16.6 sialic acids for AGP2 glycoforms. Furthermore, the majority of the proteoforms had 32 Hex and 27 HexNAc units per protein for both AGP1*F1 and AGP2.

Next, we investigated whether changes in the proteoform profiles could be observed during the pregnancy of these women. While the ratio between AGP1 and AGP2 did not greatly vary between the samples during pregnancy for women 2 and 4 (≤3% difference in abundance), the relative abundance of AGP2 was slightly higher during pregnancy for women 1 and 3 (13 and 7%, respectively). The ratio between AGP1 and AGP2 can change when the physiological states of the individuals alter (e.g., during inflammation).^32^ The glycosylation substantially changed for all of the women. The level of sialylation was higher during pregnancy compared with the postpartum samples, i.e., between 16.6 and 16.9 sialic acids per protein (Figures 4c and S9). All women had a low abundance of glycoforms with 15 sialic acids or lower (elution window between 16 and 30 min). Increased levels of sialylation during pregnancy were previously reported using complementary approaches.^14,35^ Similar to the sialylation, the branching of the glycans (increased number of HexHexNAc units) was also higher with pregnancy (Figures 4d and S9). This observation has been reported in literature for the entire pregnancy period.^15^ Finally, we compared the fucosylation level during and after pregnancy, revealing a lower abundance of the nonfucosylated glycoforms for women 1, 2, and 4, but this was not the case for woman 3 (Figure S9). Longitudinal data have shown that the decrease in the degree of fucosylation of AGP during pregnancy occurs from week 25 onward,^15^ which is within the second trimester. Therefore, it could be the case that the fucosylation level of woman 3 could still decrease within the remaining time in the second or third trimester. Altogether, information about pregnancy-associated changes regarding the abundance of genetic variants and glycosylation (sialylation, branching, and fucosylation) could be retrieved from the AEX–MS data.

## Conclusions

In this study, we developed intact AEX–MS methods to characterize AGP proteoforms. For the separation of AGP proteoforms, it was essential to use an AEX method with a salt-mediated pH gradient, providing a charge-based separation based on the number of sialic acids. This separation was key to allow discrimination between species with one additional sialic acid or two fucoses, which are very close in mass, advancing current state-of-the-art methods for AGP proteoform characterization. To annotate the intact mass spectra, comprising hundredths of signals, the analysis was complemented by glycopeptide analysis to determine the microheterogeneity and analysis of sialidase-treated AGP to obtain information on branching (number of HexHexNAc) and fucoses per protein. Combining all of the information allowed the assignment of over 400 different proteoforms in a pooled AGP sample. The intact AEX method was applied to investigate the pregnancy-associated changes in AGP profiles from four women, highlighting similar AGP glycosylation for all women during and after pregnancy. Clear pregnancy-associated AGP proteoform changes were observed for all of the investigated women. The level of both sialylation and branching was increased during pregnancy, whereas the level of fucosylation was slightly reduced.

Altogether, our novel AEX–MS approach provides an incredible amount of information on this complex protein, opening the possibility to perform comprehensive proteoform characterization. We showed that our method allows the comparison of glycosylation in a genetic variant-specific manner, adding layers of information that can often not be obtained with current analytical methods. In the future, this methodology can be applied to perform analysis of AGP proteoforms in the context of different diseases. Although the MS instrumentation employed in this study (15T FTICR-MS) may not be available in many laboratories, alternative ESI-MS systems providing good ionization conditions and a mass range for native proteins (such as Orbitrap systems) could also be used. Finally, the AEX–MS method can be expanded for the characterization of other complex plasma proteins with heterogeneous highly sialylated glycosylation.

## Data Availability

The raw mass spectrometry data is available at https://doi.org/10.5281/zenodo.11130595.

## Supporting Information Available

Assigned glycoforms of AGP after sialidase treatment measured with AEX–MS; assigned glycoforms of the intact AGP standard measured with AEX–MS; and assigned intact AGP glycoforms during and after pregnancy of four different women (XLSX)
Sequence of alpha-1-acid genetic variants; AEX method development of AGP; effect of DEN gas on ionization of AGP; glycopeptide analysis of AGP; AEX-UV of sialidase-treated AGP; EICs illustrating quantification procedure; comparison of the AGP standard before and after capturing; AEX separation of captured AGP during pregnancy; and relative quantification of AGP from (pregnant) women (PDF)
