# Supplementary material for: Anion Exchange Chromatography–Mass Spectrometry to Characterize Proteoforms of Alpha-1-Acid Glycoprotein during and after Pregnancy
Source: J Proteome Res. 2024 Jun 19;23(7):2431–40. doi: 10.1021/acs.jproteome.4c00107 (PMC11232096; doi:10.1021/acs.jproteome.4c00107)
Supplement: Supplementary file 2 — pr4c00107_si_002.pdf [file pr4c00107_si_002.pdf]

Supplementary information

Anion exchange chromatography-mass spectrometry to characterize proteoforms of alpha-1-acid glycoprotein during and after pregnancy

Guusje van Schaick [1], Manfred Wuhrer [1], Constantin Blöchl [1], Radboud J.E.M. Dolhain[2] and Elena Dominguez-Vega [1,*]

[1] Leiden University Medical Center, Center for Proteomics and Metabolomics, Leiden, the Netherlands

[2] Erasmus Medical Center, Department of Rheumatology, Rotterdam, the Netherlands

***** Correspondence: E.Dominguez_Vega@lumc.nl

Table of contents | | **Page**

**Figure S1**	Sequence of alpha-1-acid genetic variants	S2
**Figure S2**	AEX method development of AGP	S3
**Figure S3**	Effect of DEN gas on ionization of AGP	S4
**Figure S4**	Glycopeptide analysis of AGP	S5
**Figure S5**	AEX-UV of sialidase-treated AGP	S6
**Figure S6**	EICs illustrating quantification procedure	S7
**Figure S7**	Comparison of AGP standard before and after capturing	S8
**Figure S8**	AEX separation of captured AGP during pregnancy	S9
**Figure S9**	Relative quantification of AGP from (pregnant) women	S10
**Table S1**	Assigned glycoforms of AGP after sialidase treatment	Excel file
**Table S2**	Assigned glycoforms of intact AGP standard	Excel file
**Table S3**	Assigned intact AGP glycoforms during and after pregnancy	Excel file

a

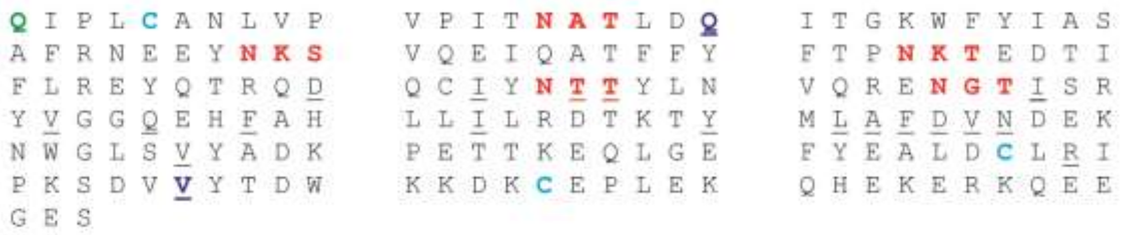

b

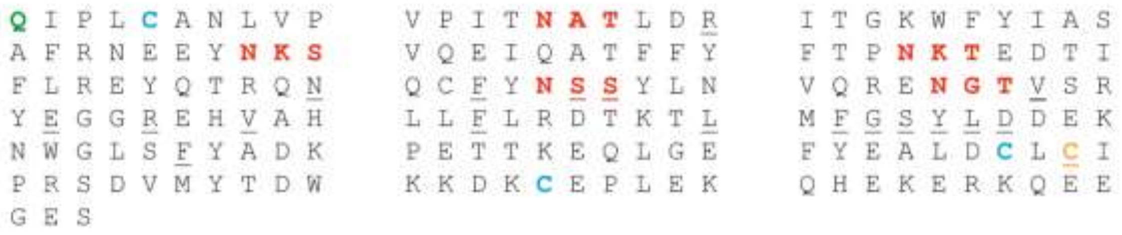

Figure S1. The sequences of the two genetic variants, that is AGP1 (**a**) and AGP2 (**b**). The underlined amino acids (in black) show the difference between AGP1 and AGP2 variants. The sites of the possible PTMs are indicated in color, including N-terminal pyroglutamic formation (green), *N*-glycosylation sites (red), cysteinylation (orange), and cysteine-forming disulfide bridges (blue). For AGP1, the sequence of the AGP1*F1 variant is presented. The amino acids that vary between the forms are indicated in dark blue, where position 38 Q → S for AGP1*S and position 174 V → M).

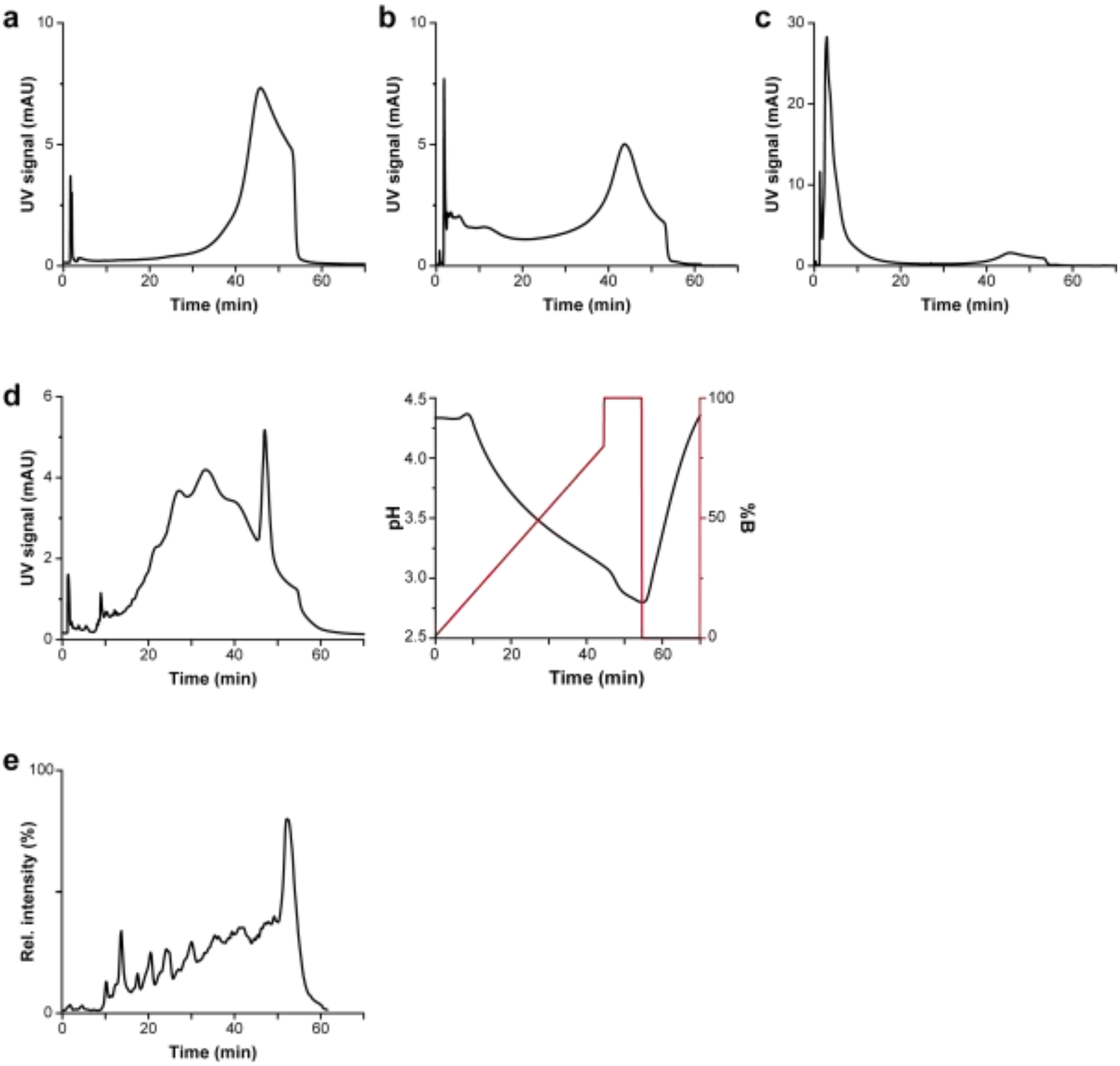

Figure S2. The AEX method was developed to separate AGP proteoforms. (**a**) AEX-UV chromatogram was obtained with mobile phases composed of 50 ammonium formate at pH 5.5 (A) and 50 mM formic acid at pH 2.5 (B). (**b**) AEX-UV chromatogram was acquired with mobile phases composed of 100 ammonium formate at pH 5.5 (A) and 100 mM formic acid at pH 2.5 (B). (**c**) AEX-UV chromatogram resulting from the use of mobile phases composed of 150 ammonium formate at pH 5.5 (A) and 150 mM formic acid at pH 2.5 (B). (**d**) The AEX-UV chromatogram (left) and measured pH gradient (right) of the optimized method using 50 ammonium formate at pH 5.5 (A) and 200 mM formic acid at pH 2.5 (B) as mobile phases. The gradient increased from 0 to 80%B in 45 min. Besides the measured pH gradient, also the programmed pH gradient is depicted (in red). (**e**) Base peak chromatogram (BPC) of AGP measured using a flow splitter and DEN gas to improve ionization.

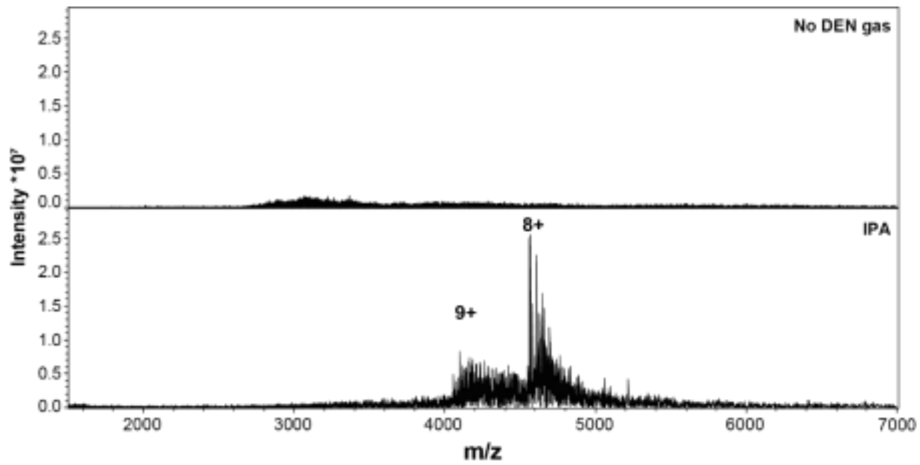

Figure S3. Mass spectra were obtained by direct infusion of AGP standard material using no dopant-enriched nitrogen gas (upper spectrum) or isopropanol (IPA)-enriched nitrogen gas (lower spectrum). When possible, the charge states are indicated in the mass spectrum.

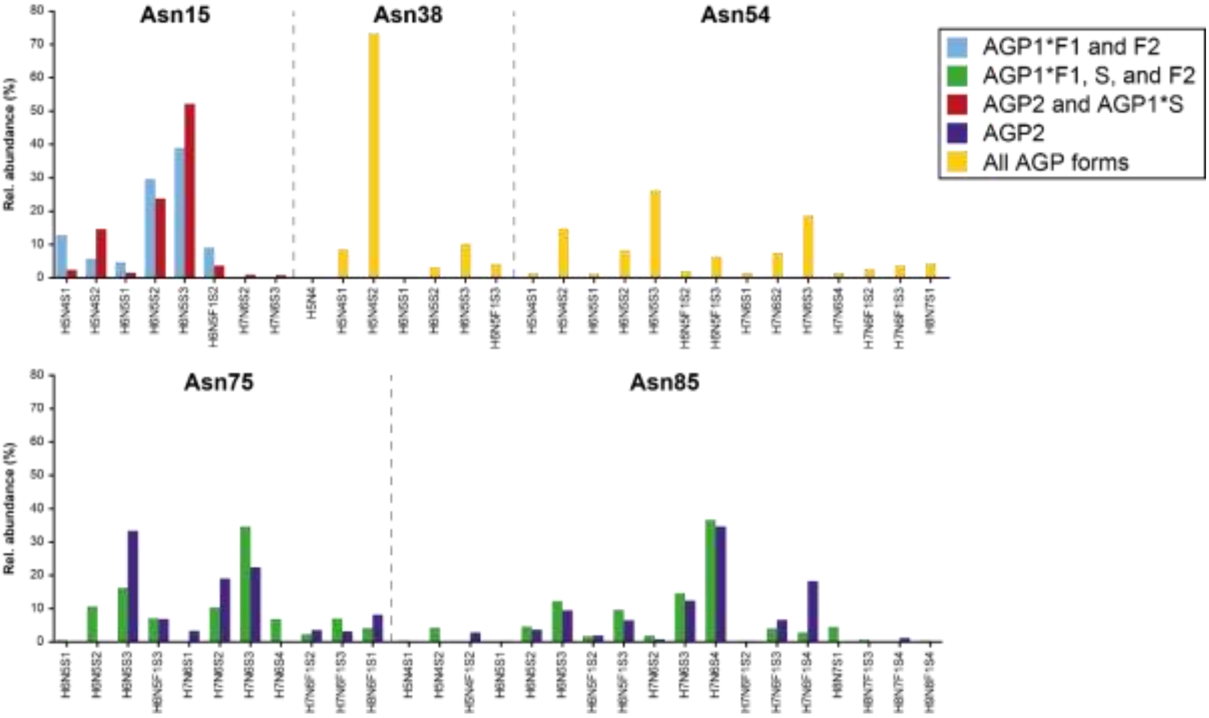

Figure S4. Glycopeptide profiling of AGP. The different glycans expressed as the relative abundance for the five *N*-glycosylation sites, that is Asn15, Asn38, Asn54, Asn75, and Asn85. The different colors of the bars indicate similar sequences for different genetic variants.

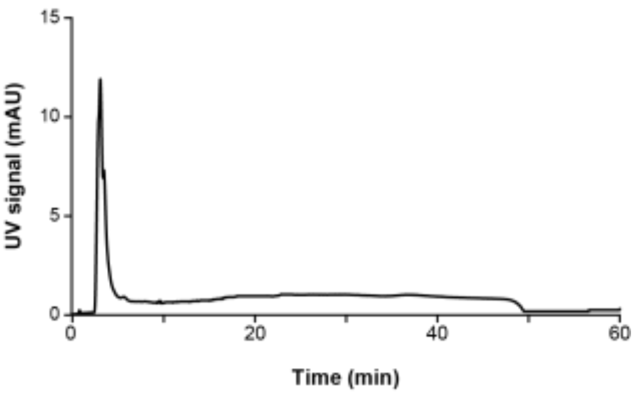

Figure S5. AEX-UV chromatogram from the AGP material treated with a sialidase. The optimal mobile phases for the complete AGP were used composed of 50 ammonium formate at pH 5.5 (A) and 200 mM formic acid at pH 2.5 (B).

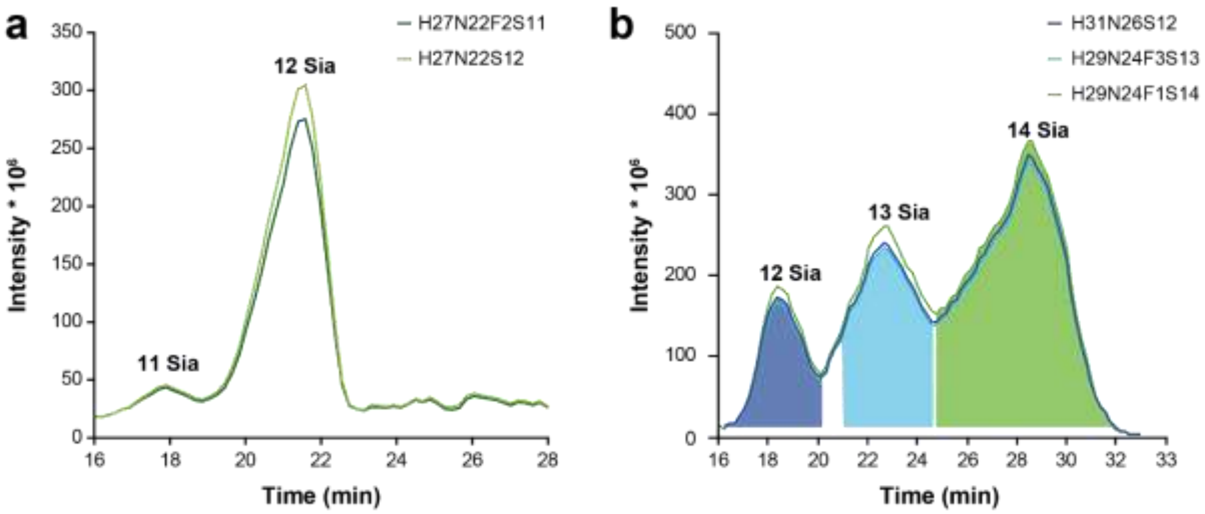

Figure S6. Extracted ion chromatograms (EICs) to illustrate the quantification procedure in Skyline. (**a**) EICs of the glycoforms H27N22F2S11 (dark green) and H27H22S12 (light green). (**b**) EICs of the glycoforms H31N26S12 (dark blue), H29N24F3S13 (light blue), and H29N24F1S14 (green).

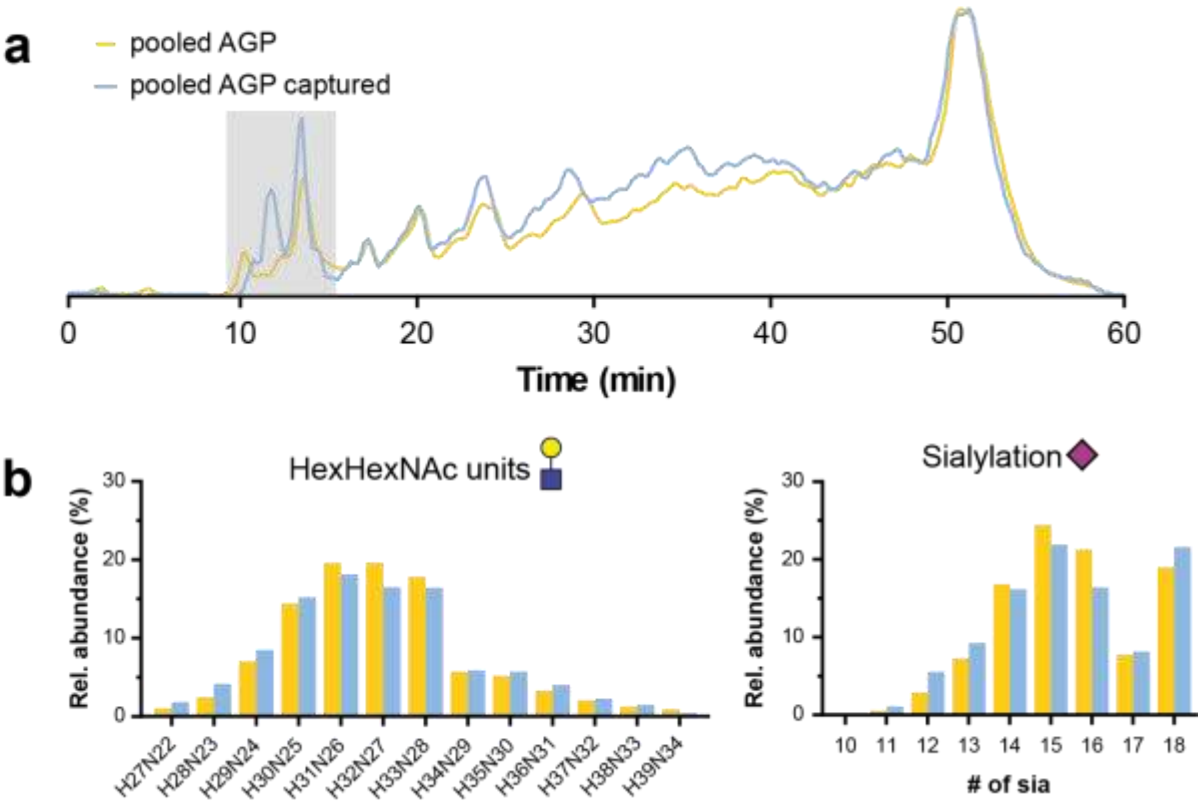

Figure S7. The AEX separation profiles and relative quantification of the AGP standard before (orange) and after capturing (blue). (**a**) The BPCs of both samples, where the indicated gray area did not contain AGP proteoforms. (**b**) Comparison of the number of HexHexNAc units (left) and the level of sialylation (right) for the AGP1*F1 variant, where only the non-fucosylated glycoforms were selected.

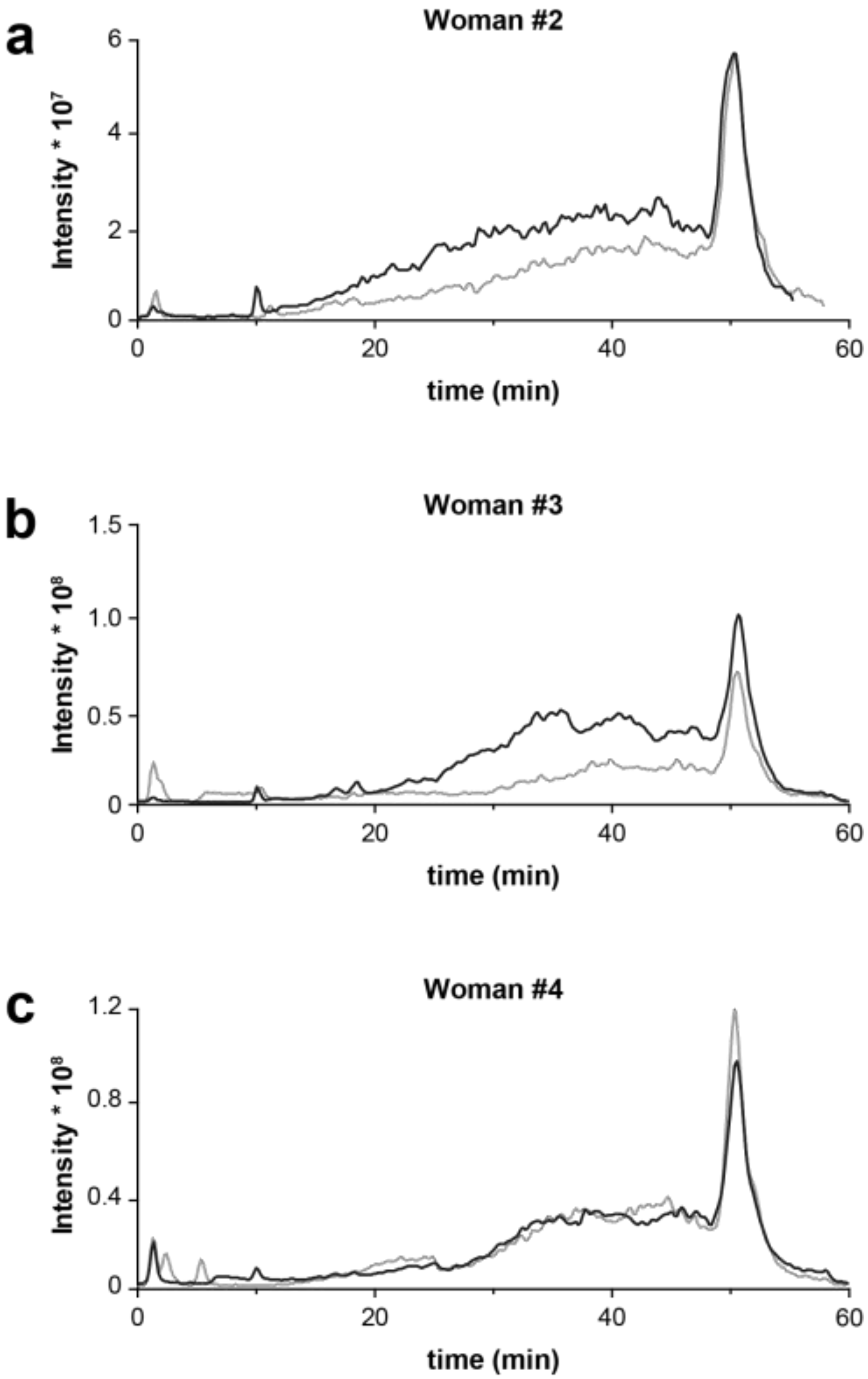

Figure S8. BPCs of the AEX separation of captured AGP during pregnancy (gray) and after pregnancy (black) from the different women (that is, woman #2, #3, and #4). The BPCs of woman #1 are displayed in the main text.

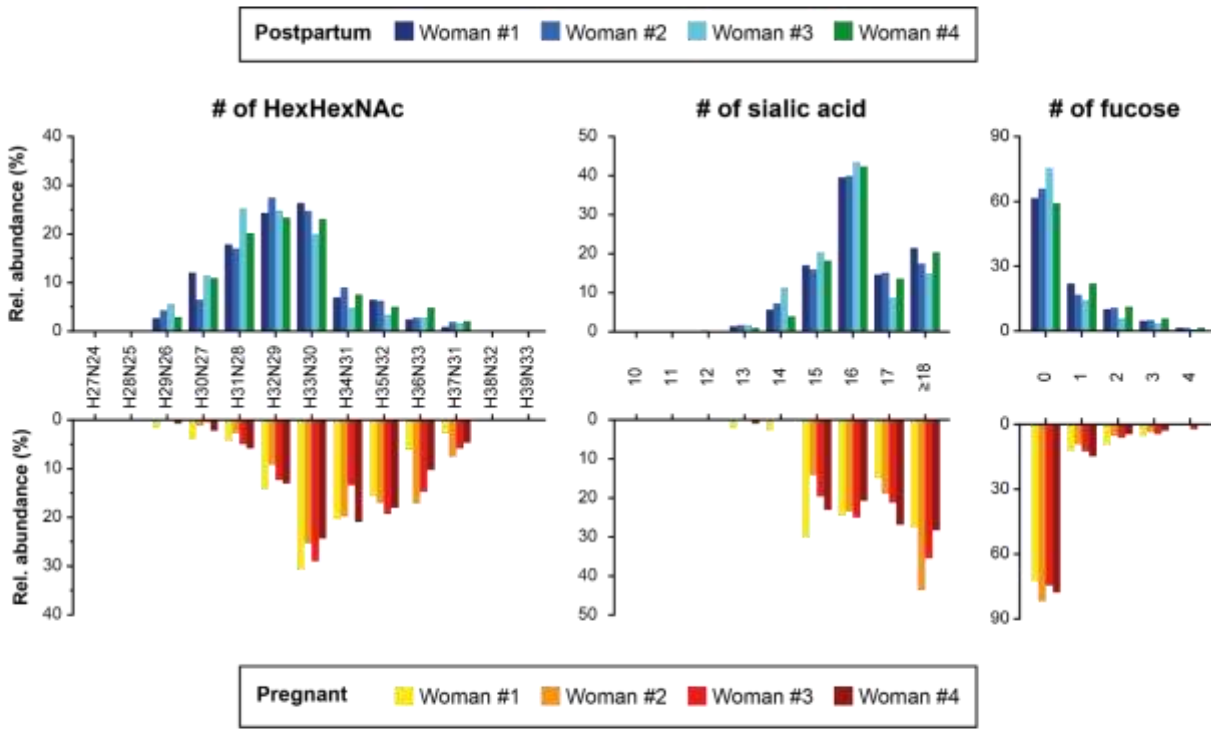

Figure S9. The relative quantification of the assigned proteoforms of the AGP captured from plasma from women during and after their pregnancy. An overview of the number of HexHexNAc, level of sialylation, and degree of fucosylation were determined for the AGP1*F1 variant. The complete list of assigned glycoforms, including their abundances) can be found in **Table S3**.
